# An exosomal strategy for targeting cancer-associated fibroblasts mediated tumors desmoplastic microenvironments

**DOI:** 10.1186/s12951-024-02452-1

**Published:** 2024-04-21

**Authors:** Xiaoxia Xue, Xiangpeng Wang, Mingshi Pang, Liuchunyang Yu, Jinxiu Qian, Xiaoyu Li, Meng Tian, Cheng Lu, Cheng Xiao, Yuanyan Liu

**Affiliations:** 1 School of Chinese Materia Medica, Beijing University of Chinese Medicine, Beijing, 100029 China; 2 Institute of Basic Research in Clinical Medicine, China Academy of Chinese Medical Sciences, Beijing, 100700 China; 3 Institute of Clinical Medicine, China-Japan Friendship Hospital, Beijing, 100029 China

**Keywords:** Desmoplastic tumors, Cancer-associated fibroblasts, Engineered exosomes, Drug delivery, Theranostic

## Abstract

Tumors desmoplastic microenvironments are characterized by abundant stromal cells and extracellular matrix (ECM) deposition. Cancer-associated fibroblasts (CAFs), as the most abundant of all stromal cells, play significant role in mediating microenvironments, which not only remodel ECM to establish unique pathological barriers to hinder drug delivery in desmoplastic tumors, but also talk with immune cells and cancer cells to promote immunosuppression and cancer stem cells-mediated drug resistance. Thus, CAFs mediated desmoplastic microenvironments will be emerging as promising strategy to treat desmoplastic tumors. However, due to the complexity of microenvironments and the heterogeneity of CAFs in such tumors, an effective deliver system should be fully considered when designing the strategy of targeting CAFs mediated microenvironments. Engineered exosomes own powerful intercellular communication, cargoes delivery, penetration and targeted property of desired sites, which endow them with powerful theranostic potential in desmoplastic tumors. Here, we illustrate the significance of CAFs in tumors desmoplastic microenvironments and the theranostic potential of engineered exosomes targeting CAFs mediated desmoplastic microenvironments in next generation personalized nano-drugs development.

**Graphical Abstract:**

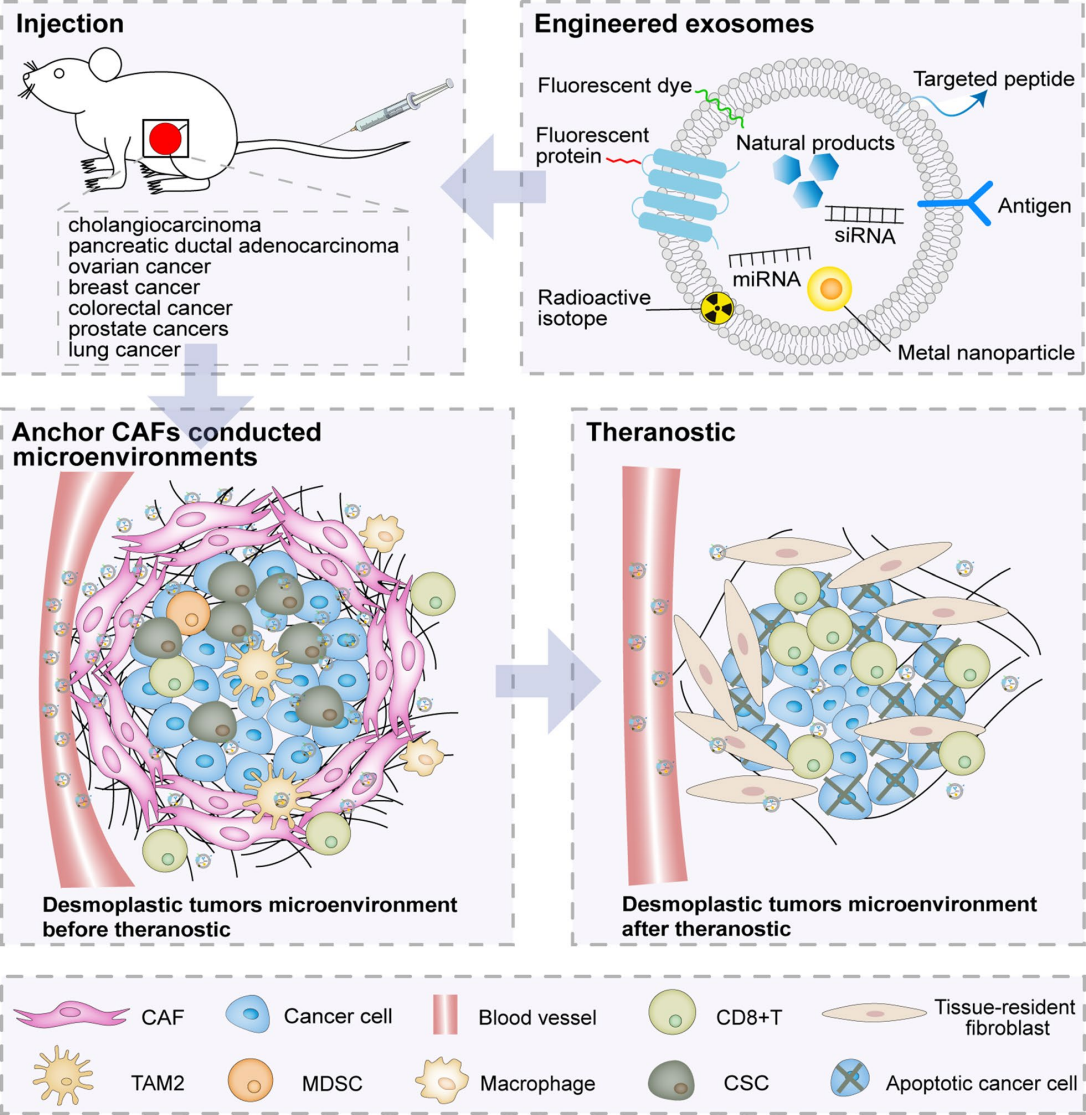

## Introduction

Despite the rapid development of delivery platforms and theranostic tools, malignancies are still one of the leading causes of human death globally. Currently, most clinically targeted-therapies mainly target cancer cells, while ignoring their surrounding substances in microenvironments. The microenvironments mainly contain stromal cells (e.g., cancer-associated fibroblasts (CAFs), immune cells, pericytes and endothelial cells), cancer cells, cytokines, chemokines, extracellular matrix (ECM) and vasculature [1]. In malignant tumors, the microenvironments of desmoplastic tumors (e.g., cholangiocarcinoma, pancreatic ductal adenocarcinoma, ovarian cancer, breast cancer, colorectal cancer, prostate cancers and lung cancer) are especially complex composed of abundant stromal cells and deposited ECM, which help establish pathological barrier to hinder the effective transport of therapeutic agents to the cancer site [2–11]. Meanwhile, the barrier also can compress blood vessels to reduce blood perfusion, further decreasing the delivery and extravasation of immune cells and therapeutic agents. Thus, aiming at the treatment of such tumors, targeting the complex tumors desmoplastic microenvironments are promising therapeutic avenues.

Notably, CAFs are the most abundant of all stromal cells in desmoplastic microenvironments, which contribute to 60–90% ECM proteins and remodel ECM through exerting a physical force on cancer cells as well as depositing, crosslinking and degrading the ECM proteins [1, 12, 13]. The ECM after CAFs remodeling is rigid, which not only serves as pathological barrier to hinder the effective delivery of drug and immune cells infiltration, but also offers scaffold for the migration and invasion of tumor cells [5, 14]. Additionally, CAFs can secret abundant cytokines and ECM proteins that activate associated signaling pathways to talk with immune cells and cancer cells, promoting immune evasion of tumor cells and cancer stem cells (CSCs)-mediated resistance of therapeutic agents [1]. Therefore, CAFs play significant role in mediating the formation of complex tumor microenvironments.

Currently, the therapies of targeting CAFs mediated desmoplastic microenvironments in most of tumors are mainly performed by synthesized nanoparticles (e.g., liposomes, micelles and polymer-based synthetic nanoparticles) that can improve the solubility, efficiency, blood circulation time of chemotherapeutic agents [2]. However, desmoplastic environment presents unique and complex pathological abnormality that limit drug delivery even in nanoscale, so there is a need to a tailored targeted-delivery system for desmoplastic tumors that can penetrate pathological barrier and may be less likely to trigger an immune response. Compared with synthesized nanoparticles, exosomes originate from biological systems can inherit abundant information (e.g., nucleic acids, lipids and proteins) from the patient cells and own good biocompatibility, biodegradability, intercellular communication and low immunogenicity, which endow them with unprecedented potential as carriers for drug delivery [15–17]. Additionally, exosomes also exhibit excellent deep penetration due to their specific phospholipid bilayer structure and naturally small size, the mediation of transcytosis, and the carriers with ECM remodeling components, which are suitable for the treatment of desmoplastic tumors [18–20]. To gain multiple functional properties, engineered modifications can be flexibly exerted on outer membrane or inner cargoes of exosomes, further improving their specific targeting and accumulation of at desired sites [15, 21, 22]. Meanwhile, with the rapid development of engineered exosomes as communicator or messenger in surrounding cells and substances have endowed them with powerful potential in the theranostic of desmoplastic tumors.

In this review, we illustrate how CAFs can be significant role in tumors desmoplastic microenvironments and their detailed mechanism, while also elaborate that engineered exosomes could target CAFs mediated desmoplastic microenvironment for the effectively delivery and theranostic of such complex tumor in the future.

### The significance of CAFs in tumors desmoplastic microenvironments

#### How CAFs constitute the complexity of desmoplastic microenvironment

Desmoplastic tumors with high-grade malignancy are characterized by fibrotic stroma and accompanied by abundant stromal cells and ECM deposition [2–4], including cholangiocarcinoma, pancreatic ductal adenocarcinoma, ovarian cancer, breast cancer, colorectal cancer, prostate cancers and lung cancer [5–11]. The dense stroma can reduce blood perfusion by compressing blood vessels, further decreasing the effective delivery and extravasation of therapeutic agents. Additionally, the surrounding substances of desmoplastic tumors establish a pathological barrier that hinders the effective transport of therapeutic agents to the cancer site, leading to the poor distribution and penetration in desmoplastic tumors [2, 3]. Thus, chemotherapy and nanomedicine therapy failed more than once to treat desmoplastic tumors even though they are sufficient to eliminate tumor cells in vitro experiments.

**Fig. 1:**
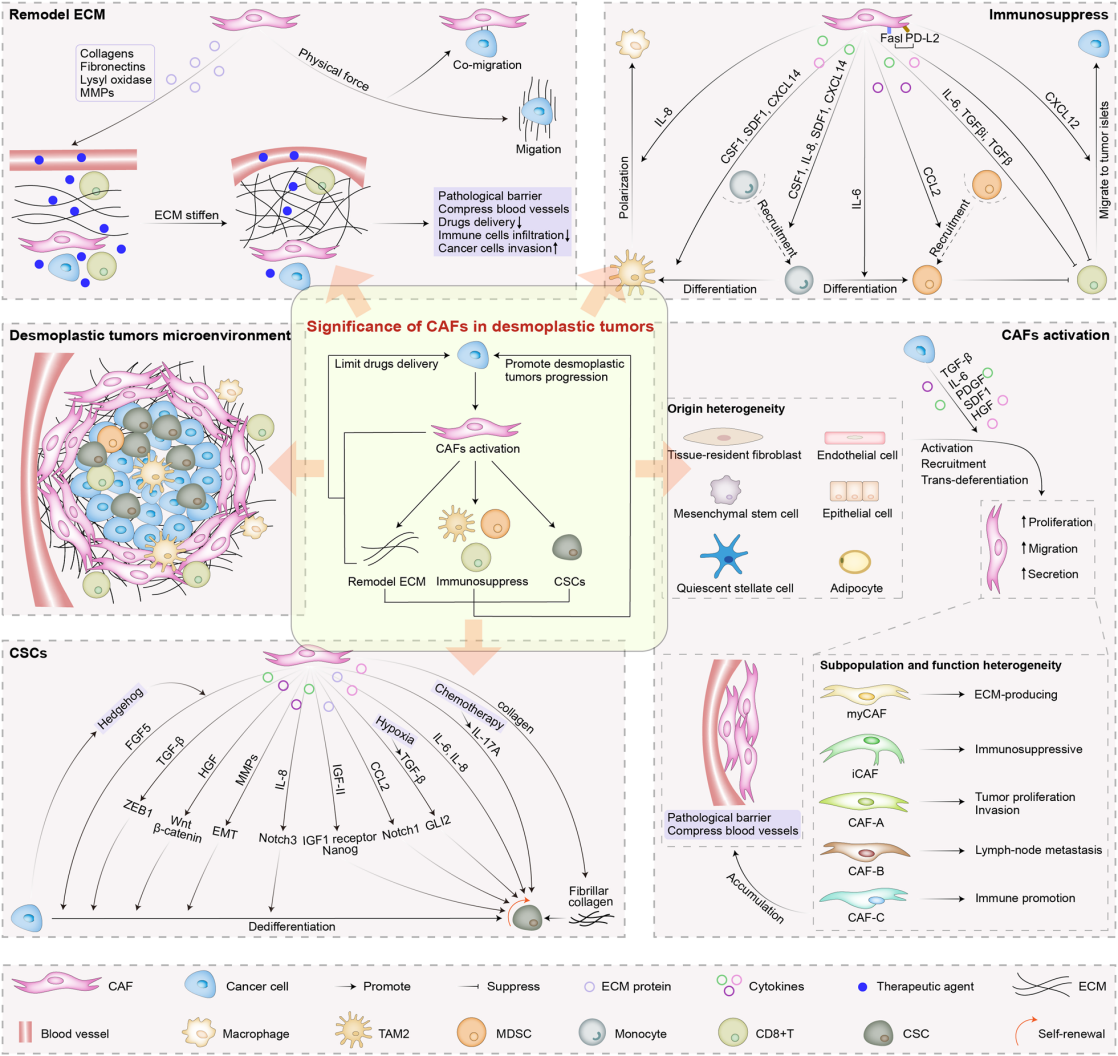
The significance of CAFs in desmoplastic tumors. CAFs secrete abundant ECM proteins and exert physical force to remodel ECM and establish pathological barrier that hinders drugs delivery and immune cells infiltration and promotes cancer cells invasion. CAFs also secret abundant cytokines and ECM proteins to induce immunosuppress and CSCs. Cancer cells affected by CAFs can secrete abundant cytokines to promote CAFs accumulation that form barrier and compress blood vessels. CSF1, colony-stimulating factor 1; FGF5, fibroblast growth factor 5; EMT, epithelial mesenchymal transition

The complex desmoplastic microenvironments are composed of abundant stromal cells (e.g., CAFs, infiltrating immune cells, pericytes and endothelial cells), cancer cells, cytokines, chemokines, ECM and vasculature [2, 3]. Notably, CAFs, the most abundant of all stromal cells merged in tumor tissues, are activated fibroblasts embedded within ECM during cancer development, which exhibit enhanced proliferative, migratory and secretory properties compared with quiescent fibroblasts and normal activated fibroblasts [1, 12, 23]. The most prominent feature of CAFs is that they can secrete a great deal of ECM proteins and remodel ECM via exerting a physical force as well as depositing, crosslinking and degrading ECM proteins during desmoplastic tumors progression, ultimately leading to the stiffness of ECM and tumors tissue [1, 13]. Subsequently, the CAFs constructed rigid ECM not only serves as pathological barrier to hinder drugs delivery and immune cells infiltration, but also provides a scaffold for the invasion and migration of tumor cells (Fig. 1) [5, 14]. Additionally, CAFs also secrete larger numbers of cytokines and chemokines to instigate immune cells into their friends and establish an immunosuppressive microenvironment [1]. As cancer cells’ henchmen, it’s not surprising that CAFs can talk directly with cancer cells by secreting cytokines and ECM proteins. It is reported that CAFs not only can promote the dedifferentiation of cancer cells toward CSCs, but also participate in the maintenance of CSCs self-renew, further promoting resistance of therapeutic agents [24, 25]. Notably, cancer cells affected by CAFs can secrete cytokines, which further triggering the recruitment and activation of fibroblasts, leading to the accumulation of high activity of CAFs and forming a positive feedback loop [1, 14, 26]. Meanwhile, the accumulation of abundant CAFs also can compress blood vessels and form pathological barriers to impede the effective drugs delivery to the inner of desmoplastic tumors [2]. In view of mentioned above, CAFs can mediate the formation of tumors desmoplastic microenvironments. Therefore, aiming at the treatment of such tumors, targeting CAFs mediated microenvironments are promising therapeutic avenues.

### Heterogeneity of CAFs

CAFs are heterogeneous cell populations with diverse subpopulations and functions in many desmoplastic tumor types, which might be ascribed to their various cellular origins, mainly including tissue-resident fibroblasts, mesenchymal stem cells, quiescent stellate cells, endothelial cells, epithelial cells and adipocytes (Fig. 1) [1, 27–29]. These precursor cells are activated, recruited, or trans-differentiated into CAFs under the stimuli of multiple factors in tumors desmoplastic microenvironments, including oxidative stress, local hypoxia, physical changes in the ECM, exosomes and DNA damage during radiation therapy, cancer-derived cytokines such as transforming growth factor-β (TGF-β), interleukin-6 (IL-6), platelet-derived growth factor (PDGF), stromal derived factor 1 (SDF1) and hepatocyte growth factor (HGF) [1, 26, 27, 30]. Activated CAFs can be identified by markers, including but not limited to fibroblast activation protein (FAP), PDGF receptors (PDGFRs) and alpha smooth muscle actin (αSMA) [1, 12, 30]. FAP is a serine protease with overexpression on CAFs in more than 90% of human cancer, which not only takes part in ECM remodeling, but also induces the immunosuppressive microenvironment [31–33]. PDGFRs, tyrosine kinase receptors, are mainly classified as two types-PDGFRα and PDGFRβ [34]. PDGF can recruit and activate fibroblasts into a CAF-like state to promote desmoplastic tumor growth and stimulate angiogenesis via interacting with PDGFRs [8, 34]. α-SMA, a cytoskeletal protein, is related to the secretion of TGF-β and the regulation of myofibroblast contractility, which has been served as a most frequent marker for the identification of CAFs and the prognosis factor of desmoplastic tumors [5, 35, 36]. Unfortunately, none of these markers are specifically expressed by CAFs because they are shared with at least one cell subpopulation, which also emphasizes CAFs heterogeneity that mainly exhibited by the existence of various CAF subpopulations in different desmoplastic tumor and accompanied by different markers [28].

In recent years, abundant CAF subpopulations have been identified using single-cell RNA sequencing in desmoplastic tumors and some of them exhibit different markers and biological functions (Table 1). In pancreatic ductal adenocarcinoma, researchers first identified two CAF subpopulations derived from pancreatic stellate cells, myofibroblast CAF and inflammatory CAF, which can dynamically reverse between them in vitro [37]. Among them, myofibroblast CAF expresses high level of αSMA and associated with an ECM-producing, conversely, inflammatory CAF expresses low level of αSMA, which increasingly secretes inflammatory factors (such as IL-6 IL-8, IL-11 and LIF) and characterizes by an inflammatory phenotype. Subsequently, these two CAF subpopulations also were found in breast cancer and cholangiocarcinoma patient samples [38, 39]. Four CAFs subpopulations (CAF-A to CAF-D) were identified in human pancreatic ductal adenocarcinoma with different biomarkers, in which periostin was a biomarker for subtype A and associated with tumor invasion and shorter survival, myosin-11 was biomarker for subtype B and related to lymph-node metastasis, podoplanin was biomarker for subtype C and associated with immune promotion and good prognosis, and no marker was selected in subtype D [40]. In breast cancer and ovarian cancer, four CAF subpopulations, CAF-S1 to CAF-S4, were identified with many markers, such as PDGFRβ, FAP, CD29, αSMA and S100A4 [35, 41, 42]. CAF-S1 and CAF-S4 subpopulations exhibited pro-invasive characteristics and accompanied with high expression of αSMA. Among them, CAF-S1 promotes cancer cell invasion mainly through secreting TGF-β and C-X-C chemokine ligan (CXCL) 12, while CAF-S4 through Notch pathway. Another difference is that in terms of immunosuppression, CAF-S1 can enhance the function of CD4^+^T and CD25^+^T lymphocytes and increase the capacity of Treg Cell, while CAF-S4 does not have these characteristics. In breast cancer, Brechbuhl et al. reported two CAF subtypes, CD146^pos^CAFs and CD146^neg^CAFs. Among them, CD146^pos^CAFs promote oestrogen-dependent proliferation and tamoxifen sensitivity of cancer cells, whereas CD146^neg^CAFs suppress oestrogen receptor expression and enhance tamoxifen resistance [43]. These examples, and many more (Table 1), are leading us to reveal the significant relationship between CAF subpopulations and desmoplastic tumors progression.

**Table 1:** CAF subpopulations and markers were identified in different desmoplastic tumors

Desmoplastic tumors	Samples	CAF subpopulations	Markers	Functions	References
Breast cancer	Patient samples	myCAF	FAP, ACTA2 and PDPN	ECM-producing	[38]
		iCAF	CXCL12	Immune evasion	
Breast cancer and ovarian cancer	Patient samples	CAF-S1	FAP, αSMA	Immunosuppressive Tumor invasion Lymph-nodes metastasis	[35, 41, 42]
		CAF-S2	Not reported	Not reported	
		CAF-S3	PDGFRβ, FSP1 and CD29	Not reported	
		CAF-S4	αSMA, CD29	Lymph-nodes metastasis Tumor invasion	
Breast cancer	Patient samples	CD146^pos^CAFs	CD146^pos^	Sustains estrogen-dependent proliferation	[43]
		CD146^neg^CAFs	CD146^neg^	Enhance tamoxifen resistance	
Breast cancer and lung cancer	Patient samples	CD10^+^GPR77^+^CAF	CD10 and GPR77	Promote tumor formation Sustain CSCs	[45]
Lung cancer	Patient samples	5 clusters	αSMA (cluster 2)	Angiogenesis	[46]
Pancreatic ductal adenocarcinoma	Patient samples; KPC mice tumors	myCAF	αSMA, transgelin, TPM1, TPM2, MMP11, POSTN and homeobox transcription factor	ECM-producing	[37, 47, 48]
		iCAF	IL-6, IL-8, IL-11, Lif, CXCL12, PDGFRα, HAS1 and HAS2	Immunosuppressive/tumor promoting	
		Antigen-presenting CAF	MHC II, CD74 and serum amyloid A3	Antigen-present Immunomodulatory	
Pancreatic ductal adenocarcinoma	Patient samples	CAF-A	POSTN	Tumor proliferation Tumor invasion	[40]
		CAF-B	Myosin-11	Lymph-node metastasis	
		CAF-C	PDPN	Promote immune	
		CAF-D	Not reported	Not reported	
Colorectal cancer	Patient samples	CAF-A	COL1A2, MMP2 and DCN	ECM-producing	[49]
		CAF-B	PDGFA, TAGLN and ACTA2	Not reported	
Cholangiocarcinoma	Patient samples; KRAS/p19 and YaP/AKT mouse tumors	myCAF	COL1A1, HAS2/HA	ECM-producing	[39]
		iCAF	HGF	Tumor promoting	
		PF/mesCAF	PF/mesothelial	Not reported	

The above studies showed that in addition to the majority of CAF subpopulations exert tumor-promoting functions, there are also a few tumor-repressing CAF subpopulations in certain cancers. Under the circumstances, nonselective targeting of CAF subpopulations may be off-target or even adverse clinical outcomes in cancer treatment. Such as, the depletion of αSMA^+^ myofibroblasts in pancreatic ductal adenocarcinoma can suppress immune surveillance with increased CD4^+^Foxp3^+^ Tregs, further reducing survival of patients [44]. Consequently, it is critical to identify more specific markers to distinguish tumor-promoting and tumor-repressing CAF subpopulations and design specific targeted-exosomes to treat desmoplastic tumors in the future. But here, the functions of tumor-promoting CAFs in tumors desmoplastic microenvironment were emphasized.

Briefly, CAFs paly significant role in mediating the formation of tumors desmoplastic microenvironment, resulting in an abundance of dense and fibrous tissue that acts as pathological barrier, which limit the efficacy of drugs by blocking the deep delivery to tumor sites. Clinically, desmoplastic tumors with high mortality present a great challenge for recurrence and metastasis. Thus, there is a need for a biocompatible targeted drug delivery system that can penetrate pathological barrier, further reducing mortality of desmoplastic tumors.

myCAFs myofibroblast CAFs, iCAFs inflammatory CAFs, Lox lysyl oxidase, PDPN podoplanin, TPM tropomyosins 1, POSTN periostin, MMP matrix metallopeptidase, HAS hyaluronan synthases, MHC II major histocompatibility complex class II

### Functions of CAFs in desmoplastic tumors progression

In desmoplastic tumor progression, CAFs secrete abundant ECM proteins and exert physical force to remodel ECM and establish pathological barrier that hinders drugs delivery and immune cells infiltration and promotes cancer cells invasion. Furthermore, CAFs also secret abundant cytokines and ECM proteins to talk with immune cells and cancer cells, inducing immunosuppression and promoting CSCs-mediated resistance for therapeutic agents (Fig. 1).

### CAFs remodel ECM

ECM, a non-cellular component, consists of macromolecules including collagen, elastin, fibrin and proteoglycan, which is a significant supporter of the tumor tissues and stromal cells in desmoplastic tumor. During desmoplasia, CAFs can secrete abundant ECM proteins and remodel ECM via exerting a physical force on cancer cells as well as continuously depositing, crosslinking and degrading the ECM proteins, resulting in the stiffness of desmoplastic tumor tissue and matricellular fibrosis that obstruct drugs delivery and immune cells infiltration and provide scaffold for the invasion and migration of tumor cells [1].

ECM remodeling also can be achieved by CAFs-mediated physical force, further promoting the invasion of tumor cells. Study reported that CAFs not only produce rich fibronectin, but also modulate fibronectin matrix by increasing contractility and traction forces that are exerted by PDGFRα and nonmuscle myosin II [50]. Then the contractile and tractive forces are transmitted to fibronectin via α5β1 integrin. Finally, CAFs organize fibronectin as parallel fibers, further promoting directional migration of prostate tumor cells. Additionally, by cell–cell contact, CAFs also exert physical forces to promote the joint invasion of CAFs and cancer cells, further remodeling ECM. The published study showed that force transmission was exerted through heterophilic adhesion between N-cadherin on CAFs and E-cadherin on tumor cells [51].

CAFs can deposit ECM proteins, such as I, III, IV and V types of collagens, fibronectins, hyaluronan, laminins, glycoproteins and proteoglycans, which contribute to the stiffness of ECM and go on as pathological barrier that hinders drugs delivery and the infiltration of immune cells [1, 52]. Pietilä et al. demonstrated that increased ECM stiffness can protect ovarian cancer cells from cisplatin mediated apoptosis via focal adhesion kinases and yes-associated protein signaling pathway [7]. The process of deposition often matches with matrix crosslinking enzymes. For instance, lysyl oxidase-like 2 is responsible for crosslinking collagen and elastin leading to the stiffness of ECM, which can be used as a biomarker of poor prognosis in cholangiocarcinoma and pancreatic ductal adenocarcinoma [5, 53]. Intriguingly, instead of isolating cancer cells, the barrier for ECM stiffening facilitates their invasion and spread [7, 54]. The overexpression of FAP in pancreatic cancer CAF can remodel ECM through modulating fibronectin levels and increasing the organization of collagen fiber, further forming a hardened and parallel fiber that increases the directionality of tumor cell invasion [31]. Simultaneously, in the process of desmoplasia, CAFs produce ECM-degrading proteases, such as MMPs, which can remodel ECM and promote the metastasis and invasion of tumor cells. The overexpressed MMPs (such as MMP-2, MMP7, MMP-9, MMP-11 and MMP-14) are frequently induced by TGF-α/β, NF-κB and WNT, which are related to poor prognosis for cholangiocarcinoma and breast cancer [5, 55, 56]. Therefore, desmoplasia is a dynamic stromal alteration process via CAFs-mediated local remodeling of ECM.

### CAFs induce immunosuppression

Except for the pathological barrier caused by CAFs and ECM to hinder immune cells infiltration, CAFs can secrete larger numbers of cytokines and chemokines to recruit immunosuppressive cells or reduce the activities of immune effector cells, promoting the immune escape of cancer cells [1, 27, 57].

In desmoplastic tumors progression, the most common immunosuppressive cells, such as tumor associated macrophages (TAM) 2 and myeloid-derived suppressor cells (MDSCs), are related to poor clinical prognosis of patients [58, 59]. TAMs are conventionally divided into two subpopulations according to the differentiation degree and function, TAM1 and TAM2, in which TAM1 primarily plays an antitumor role with proinflammatory properties, whereas TAM2 exhibits tumor-promoting activity [59, 60]. CAFs-derived colony-stimulating factor 1 can recruit monocyte and trans-differentiation toward the TAM2 via the interaction of colony-stimulating factor 1 and its receptor to promote cancer progression [61]. IL-8 secreted from CAFs can attract monocytes and subsequently facilitate the polarization of macrophages into TAM2, which inhibits the functioning of natural killer cells in colorectal cancer [62]. Reciprocally, TAM2 recruited by CAFs can also further stimulate CAFs activation and progression. For instance, CAFs-derived SDF1 and CXCL14 can promote monocyte recruitment and trans-differentiation toward the TAM2, in turn, TAM2 can secrete SDF-1 and IL-6 to activate CAFs, further making up a positive loop that facilitates immunosuppression and prostate cancers progression [63, 64]. FAP^+^CAFs-derived C–C chemokine ligand (CCL) 2 can mediate the recruitment of MDSCs through the FAP-STAT3-CCL2 pathway, further establishing an immunosuppressive environment [32]. Subsequently, a similar act of CAF-derived CCL2 on MDSCs was discovered in lung cancer, and the accumulation of MDSCs suppressed CD8 + T cell function [65]. Additionally, CAFs also secrete SDF-1 recruit monocytes and trigger their differentiation into MDSCs via IL-6/STAT3, further inhibiting T cell proliferation [66].

Cytotoxic T lymphocytes (CTLs, also called CD8^+^T) mainly induce cytotoxic activities to promote the apoptosis of cancer cells, which are taken as significant component of antitumor effects [59, 67]. However, the existence of CAFs could inhibit the growth, infiltration and antitumor immunity of CD8^+^T cells [68]. CXCL12 is upregulated in activated pancreatic stellate cells, which can decrease the migration of CD8^+^T cells to cancer islets in pancreatic ductal adenocarcinoma [69]. CAFs can also secrete IL-6, βig-h3 (also called TGFβi) and TGFβ to restrict the activity of CD8^+^T cell [66, 70, 71]. Additionally, CAFs also can suppress CD8^+^T cells by the activation of immune checkpoint. Lakins et al. [72] reported that CAFs participate in sampling, process, cross-present antigen and induce CD8^+^T cells death via coincidental upregulation of FAS and PD-1 on T cells and FASL and PD-L2 on CAFs.

### CAFs promote CSCs

CSCs are special stem-like subpopulation in tumors that can self-renew and differentiate into tumor cells, leading to resistance of therapeutic agents [73, 74]. In desmoplastic tumors progression, CAFs-secreted cytokines and ECM proteins not only can promote the dedifferentiation of cancer cells toward CSCs‐like phenotype, but also participate in the maintenance of CSCs self-renewal, further promoting the resistance of therapeutic agents [24, 25].

CAFs can transform cancer cells into CSCs. Chen et al. demonstrated that differentiated lung cancer cells can dedifferentiate into CSCs when co-culturing with CAFs [75]. Under the stimulation of cancer-released hedgehog ligands, CAFs can secret fibroblast growth factor 5 to facilitate the dedifferentiation of breast cancer cells into CSCs [76]. Breast CAF-secreted TGF-β can increase ZEB1 transcription and induce the dedifferentiation of tumor cells into CSCs state [77]. Colorectal CAF-secreted HGF can promote the transformation of tumor cells into CSCs through Wnt/β-catenin signaling pathway [78]. Prostate cancer-derived IL-6 can activate fibroblast, and then activated prostate CAFs can secrete MMPs and trigger epithelial mesenchymal transition in cancer cells to facilitate the transformation into CSCs‐like phenotype [79]. In ovarian cancer, CAFs-secret IL-8 also can activate Notch signaling pathway to promote CSCs [80].

Additionally, CAFs play significant role in the maintenance of CSCs. CAFs-derived insulin-like growth factor-II (IGF-II) can activate IGF-II/IGF1 receptor/Nanog signaling to maintain CSCs, which is related to poor prognosis in lung cancer patients [75]. In breast cancer, CAFs-secreted CCL2 can induce CSCs self-renewal via Notch1 signaling [81]. Under hypoxia tumor microenvironment, colorectal CAFs-secreted TGF-β can promote CSCs maintenance and reduce sensitivity to 5-fluorouracil and oxaliplatin by hypoxia/TGF-β/GLI2 signature [82]. CD10^+^GPR77^+^ CAFs-derived IL-6 and IL-8 can induce CSCs self-renewal and enrichment, further protecting breast and lung cancer cells from neo-adjuvant chemotherapy-induced cell death [45]. In colorectal cancer, under the stimulation of clinically relevant chemotherapy agents (5-fluorouracil, oxaliplatin and leucovorin), CAF-secreted IL-17A can maintain CSCs self-renewal [83]. Furthermore, research showed that stimulated CAFs can promote the formation of fibrillar collagen that provides a supportive niche for CSCs self-renewal, further reducing the sensitivity of docetaxel chemotherapy [76].

### Engineered exosomes serve as promising drug delivery platform for targeting CAFs mediated desmoplastic microenvironments

CAFs mediated desmoplastic microenvironments have become an appealing target for treating desmoplastic tumors. Due to the existence of pathological barrier, targeting cancer cells remains challenging for clinicians. The synthesized nanoparticles (e.g., liposomes, micelles and polymer-based synthetic nanoparticles) are currently the most commonly used carriers for drug delivery, for which their features (e.g., diameter, surface charge, hydrophobicity/hydrophilicity and receptor-mediated endocytosis) can be easily adjusted or modified. Thus, synthesized nanoparticles can significantly improve the residence time, cellular internalization and release of chemotherapeutic agents [16]. However, due to the existence of unique and complex pathological barrier of desmoplastic tumors (e.g., CAFs, ECM and compressed blood vessels), which prevent some synthesized nanoparticles from going further into tumor sites, and most of which are not biodegradable leading to immunogenicity or accumulative toxicity. Compared with synthesized nanoparticles, the inherent properties of exosomes, including biocompatibility, biodegradability, intercellular communication and low immunogenicity, endow them with unprecedented potential as carriers for drug delivery [2, 15, 84]. Additionally, exosomes also exhibit excellent deep penetration due to their specific phospholipid bilayer structure and naturally small size, the mediation of transcytosis, or the carriers of ECM remodeling components, which are easier to cross ECM and, most importantly, easier fusion with receptor cells to release drugs into them [18–20]. Exosomes are also highly engineerable. To improve the targeted delivery of natural exosomes, further engineered modifications are needed to optimize them more specific targeting property. Based on the above advantages, engineered exosomes are suitable for targeting CAFs mediated tumors desmoplastic microenvironments, which could be taken as a promising drug delivery platform.

### Exosomes mediate intercellular communication

Exosomes, with small diameter of 30–200 nm and lipid bilayer membrane, are extracellular vesicles secreted by cells, which originate from membrane invaginations, and then multivesicular bodies are formed through inward budding of the endosomal membrane, finally, exosomes can be released by fusing with plasma membrane [85, 86]. Intraluminal vesicles (future exosomes) are generated by the inward budding of membrane and accumulate in multivesicular bodies, which involves cargo sorting machinery, mainly including the endosomal sorting complex required for transport (ESCRT)-dependent or ESCRT-independent [86]. The ESCRT machinery contains five subcomplexes, in which ESCRT-0 and ESCRT-l take charge of ubiquitinated proteins cluster, ESCRT-I and ESCRT-II induce bud formation, ESCRT-III drives vesicle cleavage and releases intraluminal vesicles into the endosome to form multivesicular bodies, ATPase vacuolar protein sorting gene 4 depolymerizes the polymerized ESCRT-III that allow recycling of ESCRT [87]. Additionally, endosomal sorting also can be accomplished by ESCRT-independent machinery. The published studies showed that ceramide and tetraspanin CD63 are involved in this progress [88, 89]. Finally, the multivesicular bodies with various intraluminal vesicles can either be degraded by fusing with lysosomes or be released by fusing with plasma membrane [17].

Released exosomes from donor cells express various surface molecules, including tetraspanins, lipids, lectins and integrins, which enable them to target recipient cells and deliver contents (nucleic acids, lipids and proteins) to regulate the biochemical composition and behavior of recipient cells [17, 90]. Once exosomes attach to the plasma membrane of target cells, then can activate the intracellular signaling pathway via receptor binding to elicit functional responses [17]. Exosomes also can be internalized by clathrin, caveolae and lipid rafts-mediated endocytosis or phagocytosis/macropinocytosis and then reach multivesicular bodies, which could either be degraded via lysosomes or release contents into the cytoplasm of target cell by back fusion with the membrane of multivesicular bodies [17, 21]. Additionally, exosome contents can be directly released into the cytoplasm via membrane fusion of exosomes and recipient cell [17, 91]. Thus, in the body, exosomes can be released by almost all cells and communicate with their donor cells or neighboring/distant recipient cells to regulate various physiological and pathological processes, including blood coagulation, inflammation, immune response and tumor progression, among others [92–94].

### Engineered exosomes serve as multifunctional carriers for drug delivery

Synthesized nanoparticles are currently the most commonly used carrier for drug delivery, which can improve the residence time and selectivity of chemotherapeutic agents [15]. But the clinical application of these nanoparticles has some difficulties, such as toxicity, low bioavailability of drugs and the trigger of immune response [2]. Additionally, synthesized nanoparticles rely heavily on the enhanced permeability and retention for passive targeting and accumulation, which limit drug delivery in desmoplastic tumor due to the existence of unique and complex pathological barrier [2]. Notably, the following advantages of exosomes offer the maximum possibility for solving these problems. First, exosomes originate from biological systems and could be acquired from the patient’s cells, which may be less likely to trigger an immune response than synthetic carrier [17, 94]. Additionally, CD47 on exosomal surface can protect them from macrophages-mediated phagocytosis via binding with signal regulatory protein alpha resulting in a don’t eat me signal, which prolongs circulation time of exosomes and improves therapeutic efficacy [95]. Second, exosomes have lipid bilayer membranes that can directly fuse with the membranes of target cell to improve the efficiency of drug internalization [15, 96]. Additionally, this membrane also can protect the loaded therapeutic agents from degradation, such as natural products, small interfering RNA (siRNA), microRNA (miRNA) and proteins [16]. Third, exosomes have superb deep penetration due to their specific phospholipid bilayer structure and naturally small size, the mediation of transcytosis, and the carriers with ECM remodeling components, which are easier to cross ECM barriers [18–20]. Existing reports showed that exosomes loaded with cargo can penetrate pathological barrier and improve the efficacy of therapeutic agents compared with free drug, and it has been recently revealed that the transcytosis may be associated with crossing this barrier [18, 97]. For instance, exosome carried with neuropilin-1 can penetrate blood–brain barrier and pancreatic cancer tissues through receptor-mediated transcytosis [98, 99]. Furthermore, exosomes carried ECM remodeling components can achieve deep penetration and tumor accumulation in desmoplastic tumor via degrading dense ECM. Feng et al. and Wei et al. reported that exosomes loaded with hyaluronidase PH20 and MMPs (e.g., MMP-9 and MMP-14) can penetrate dense ECM barrier via ablating them, further enhancing drug delivery and immune cells infiltration [19, 100]. Therefore, based on above advantages, exosomes as a carrier for drug delivery can carry therapeutic agents for long-time stable circulation in the body and penetrate the pathological barrier to reach the desired sites and then exert anti-tumor effects.

### Engineered exosomes can target CAFs mediated desmoplastic microenvironments

Compared with traditional nanomedicine, the homing and penetration property of exosomes endow them with the advantage of targeting recipient cells in dense desmoplastic tumors. Nevertheless, in order to increase the capacity of targeting desired sites for exosomes, further engineered modifications are necessary. The precise targeting of recipient cells is achieved mainly through the high affinity between the receptor and ligand. Thus, appropriately modified exosomes can easily target the specific sites of CAFs mediated tumors desmoplastic microenvironments, such as cancer cells, CAFs, ECM and immune cells. The following are engineering modification strategies of exosomes.

Genetic engineering is the most widely used modification strategy, which aims to fuse ligands with targeted function to transmembrane proteins (Lamp2b, CD9, CD63, CD81, glycosyl phosphatidylinositol, PDGFR, C1C2 region of lactadherin) on the exosomal surface [22, 101, 102]. The targeting peptides, such as iRGD and tLyP-1, can be fused to Lamp2b on exosomes to target cancer cells by binding to the αv integrins and neuropilin-1/2 receptors on their membranes, respectively [96, 103]. In addition to the targeting peptide, transmembrane proteins also can be fused with antibody fragments. For instance, PDGFR transmembrane region can be fused with antibodies, such as anti-human CD3 and HER2, which dually target CD3 receptor of T-cell and HER2 receptors of breast cancer [104]. The CD63/CD9/CD81, C1C2 domain of lactadherin and glycosyl phosphatidylinositol-anchored proteins on exosomes also can be engineered with different ligands to target desired cells by binding to their affinity receptors [105–107]. Additionally, parental cells of exosomes can be modified by using plasmids or viruses that encode fusion ligands on cells, and then acquire abundant exosomes with the expression of gene-associated ligands [101]. Hu et al. showed that membrane-anchored FAP can be modified on the cancer cells by a lentiviral vector [33]. Then obtained cancer-derived exosomes with the expression of FAP antigen that can target CAFs to exert anti-tumor effects. Hong et al. designed that glycosylphosphatidylinositol-anchored hyaluronidase can be modified on HEK293T cells by plasmid encoding full-length PH20, and then isolated exosomes can target CAFs-mediated overly accumulated ECM [108]. Thus, genetic engineering can select the expression of proteins on the exosome and endow them with the capacity of targeting CAFs mediated tumors desmoplastic microenvironments.

Although less explored, exosomes could also be engineered by chemical methods. Some targeted peptides are conjugated to the membrane of exosomes by the cycloaddition reaction and bio-orthogonal click reaction to target CAFs mediated desmoplastic microenvironments [101, 102]. The study reported that RGE peptide can be conjugated with the membrane of loaded-curcumin exosome by click chemistry, which can easily reach glioma by binding to neuropilin-1 to exert the imaging and therapeutic functions [99]. The CREKA peptide can target to CAF based on the high affinity between the peptide and overexpressed fibronectin [109]. Additionally, the amphipathic molecules of distearoyl phosphoethanolamine-polyethylene glycol (DSPE-PEG) can be inserted into exosomes membrane, which is another chemical method. Subsequently, some targeted molecules are conjugated to DSPE-PEG for targeting cancer or CAF sites. It is reported that RGD and folate can be conjugated to DSPE-PEG and inserted into exosomal membrane, endowing them with the ability to target cancer sites [102, 110]. Macrophage-derived exosomes with aminoethyl anisamide (AA)-DSPE-PEG can target overexpressed sigma receptor on non-small cell lung cancer [111]. Previous study revealed that sigma receptor also is overexpressed on activated CAFs and AA-DSPE-PEG can target CAFs [112]. FH peptide (FHKHKSPALSPVGGG) can be conjugated to DSPE-PEG to target overexpressed tenascin C on CAFs [113]. Chemical modification has the advantages of short time consuming, high efficiency and mild reactions.

### Theranostic potential of engineered exosomes for targeting CAFs mediated desmoplastic microenvironments

**Fig. 2:**
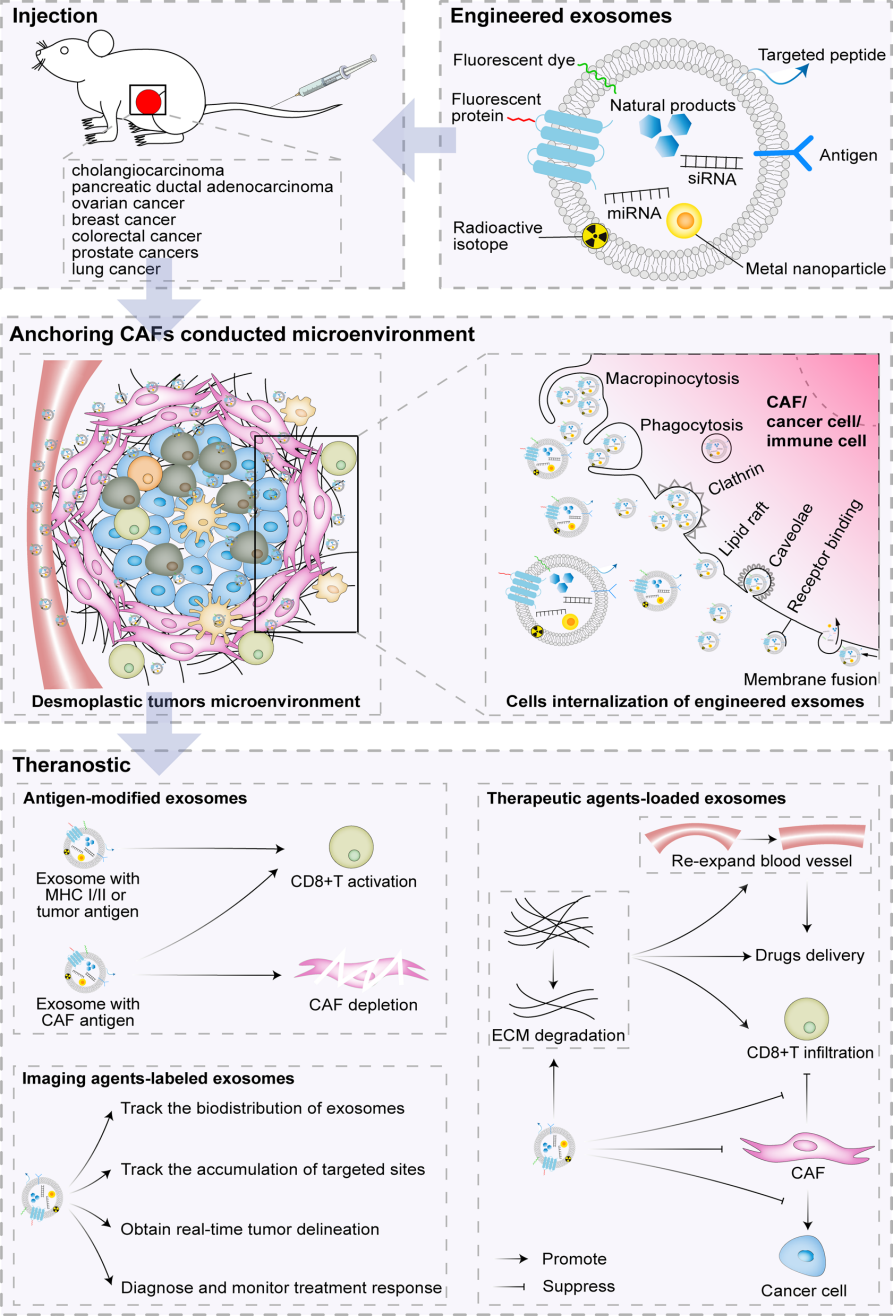
Schematic illustration of engineered exosomes targeting CAFs mediated desmoplastic microenvironments to exert theranostic potential on desmoplastic tumors models. After injection, antigen and theranostic agents-loaded exosomes can target CAFs mediated desmoplastic microenvironments to exert theranostic potential via stimulating the activation of CD8^+^T cell, removing ECM, inactivating CAFs, inhibiting the signal transduction of CAF-tumor cells and CAF-immune cells crosstalk, and imaging in vivo

Desmoplastic tumors are characterized by abundant accumulation of CAFs and ECM deposition, which surround and infiltrate the tumor cells and block the infiltration of immune cells [2–4]. Thus, targeting the main ingredients (such as CAFs, ECM, immune cells and cancer cells) in CAFs mediated desmoplastic microenvironments and influencing their behavior, might solve the current clinical and therapeutic stalemate in desmoplastic tumors. The rapid development of engineered exosomes as cargo delivery carriers have endowed them with power potential in the treatment of desmoplastic tumors (Fig. 2, Table 2). Immune cells and cancer cells-derived exosomes may serve as vehicles to trigger anti-tumor immune responses through antigen presentation. Therefore, exosomes modified appropriately with antigens may target CAFs mediated desmoplastic microenvironments to and exert anti-tumor immune effects. Except for antigenic modifications on the membrane, engineered exosomes could load therapeutic agents into their lumen and membrane, including natural products, siRNA, miRNA and proteins, which can be effectively released into CAFs mediated desmoplastic microenvironments to treat desmoplastic tumors mainly through removing ECM, inactivating CAFs and inhibiting the signal transduction of CAF-tumor cells and CAF-immune cells crosstalk. Additionally, engineered exosomes carrying imaging agents have been applied in vivo imaging to precisely track the biodistribution of therapeutics-loaded exosomes and accumulation of targeted sites and obtain real-time tumor delineation, further helping physicians to diagnose and monitor treatment response of desmoplastic tumors.

**Table 2:** Theranostic potential of engineered exosomes for targeting CAFs mediated desmoplastic microenvironments

Theranostic	Payload	Target sites	Function	Desmoplastic tumors	References
Therapy	Antigens	CTLs, CAFs	Present tumor antigens to CTLs Reprogram CAF-mediated immunosuppressive microenvironment	Breast cancer, lung cancer and colon cancer	[33, 114]
	PH20, doxorubicin	ECM	Degrade ECM Enhance the activation and infiltration of CD8^+^T cells Promote the maturation of CD103^+^ dendritic cells Enhance drug delivery	Prostate cancers and breast cancer	[19, 108, 115]
	RGD, paclitaxel	ECM, tumor cells	Remove CAFs-mediated αSMA and type 1 collagen Enhance drug delivery	Pancreatic cancer	[116]
	MMP9 and MMP14	ECM	Degrade tumor collagens Enhance the infiltration of CD8^+^T cells Anti-PD-1 antibody	Breast cancer	[100]
	miRNA-494 and miRNA-542-3p	Stromal cells	Activate MMPs and degrade ECM	Pancreatic cancer	[117]
	Silenced dicer	CAFs	Decrease FAP and α-SMA level of CAFs	Ovarian cancer	[118]
	siS100A4	CAFs	Inhibit CAF-mediated lung metastasis of breast cancer	Breast cancer	[119]
	miR-5100	CAFs	Inhibit the CXCL12/CXCR4 axis	Breast cancer	[120]
Diagnosis	PKH26, DiR, cyanine, fluorescent proteins	Tumor tissues	Fuorescence imaging	Colon cancer and prostate cancer	[121–123]
	gold-iron oxide nanoparticles s	Tumor tissues	Magnetic resonance imaging Selectively damage tumor cells via thermal ablation	Breast cancer	[124, 125]
	^99m^Tc, ^131^I and ^111^In-oxine	Tumor tissues	Single-photon emission computed tomography	Colon cancer	[121, 122]

### Therapeutic potential of antigens-modified exosomes

Immune cells and cancer cells-derived exosomes can inherit amount antigens or proteins from their donor cells, which can participate in antigen presentation and trigger CTLs to exert specific anti-tumor immune responses [126]. Therefore, exosomes themselves or exosomes modified with appropriate antigens can target CAFs mediated desmoplastic microenvironments to exert anti-tumor immune effects, further treating desmoplastic tumors.

Dendritic cells and B lymphoma cells-derived exosomes contain MHC I/II, CD86 and heat shock proteins, which can facilitate antigen presentation to prime CD4^+^T cell and stimulate the activation of CD8^+^T cell, further exerting efficient antitumor effects [126, 127]. Tumor cell-derived exosomes inherit MHC II and amount tumor-specific antigens, which can present tumor antigens to CTLs and trigger efficient antitumor effects [22, 114]. Additionally, exosomes modified with appropriate antigens also can target CAFs to reprogram CAF-mediated immunosuppressive microenvironment. Hu et al. performed specifically antigenically modified on tumor cell-derived exosomes to achieve dual targeting of cancer cells and CAF [33]. For instance, in breast, lung and colon cancer models, the FAP gene-engineered tumor-derived exosomes not only retain tumor antigen but also FAP antigen, which could induce CTLs against cancer cells and FAP^+^CAFs and reprogram immunosuppressive microenvironment by transforming TAM2 into TAM1 and reducing the infiltration of MDSCs. Furthermore, the specific anti-tumor immune responses also can facilitate cancer ferroptosis through the CTLs-derived interferon-gamma from and the depletion of FAP^+^CAFs.

### Therapeutic potential of therapeutic agents-loaded exosomes

Engineered exosomes exhibit higher biocompatibility, targeted delivery, penetration and drug protection for long-time stable circulation, which improve the effectiveness of natural products and protecting nucleic acids and proteins from degradation [15, 16]. Finally, the loaded contents can effectively exert anti-tumor effects mainly through removing ECM, inactivating CAFs and inhibiting the signal transduction of CAF-tumor cells and CAF-immune cells crosstalk.

Hyaluronan and collagen are main components of ECM secreted by CAFs, which can help to establish a pathological barrier and hinder drug delivery [1, 2, 128]. Exosomes carried with protease and ECM-associated miRNAs can penetrate dense ECM barrier via ablating them and then enhance drug delivery and immune cells infiltration. Feng et al. reported that PH20 hyaluronidase and folic acid-modified exosomes can enhance doxorubicin delivery by degrading ECM and inhibit the metastasis of breast cancer caused by hyaluronidase treatment [19]. Hong et al. designed a PH20-modified exosomes that can target CAF-mediated overly accumulated ECM and ablate them to increase immune cells infiltration and the penetration of doxorubicin in prostate cancers and re-expand the tumor blood vessel [108]. Hong et al. also reported that PH20-modified exosomes not only deeply penetrate breast cancer tissues via hyaluronan degradation, but also activate the maturation of CD103^+^ dendritic cells in vivo and CD8^+^T cells activation [115]. Al Faruque et al. designed the extracellular vesicles modified with RGD and magnetic nanoparticles and loaded with paclitaxel, which exhibit two key characteristics: first, the modified vehicles can effectively penetrate the fibrotic barrier via spontaneously removing CAFs-mediated αSMA and type 1 collagen, second, extracellular vesicles loaded with paclitaxel can be effectively internalized into pancreatic cancer cells and eventually regressing the tumors [116]. Furthermore, metalloproteinase (MMPs) was also widely reported for degrading collagen of ECM. Macrophage-derived exosomes overexpressed MMP9 and MMP14 could degrade tumor collagens, further enhancing CD8^+^ T cells infiltration and the deep delivery of anti-PD-1 antibody to exert anti-tumor effects [100]. Besides protease, ECM-associated miRNAs also could degrade ECM. Rana et al. reported that exosomes carried miRNA-494 and miRNA-542-3p could be delivered to neighboring stromal cells, further activating MMPs and degrading ECM in pancreatic cancer [117]. SiRNA can trigger the post-transcriptional gene silencing and have the potential for cancer treatment [129]. Engineered exosomes with gene silencing can inactivate CAFs and inhibit the signal transduction of CAF-tumor cells to exert anti-tumor effects. Li et al. reported that exosomes with silenced dicer can significantly decrease FAP and α-SMA level of CAFs in ovarian carcinoma [118]. The upregulation of S100A4 in CAFs is associated with tumor metastasis [1]. Zhao et al. designed the cationic bovine serum albumin-siS100A4-loaded exosomes that can trigger the post-transcriptional gene silencing and further inhibit the CAF-mediated lung metastasis of breast cancer [119]. Additionally, engineered exosomes also can inhibit the signal transduction of CAFs-immune cells. The main source of CXCL12 is FAP^+^CAFs that can induce immunosuppression through CXCL12/CXCR4 axis [130]. In breast cancer, Yue et al. designed the miR-5100-load exosomes that can inhibit the CXCL12/CXCR4 axis, further suppressing the invasion of tumor cells [120].

### Theranostic potential of imaging agents-labeled exosomes

Exosomes carried with imaging agents can be applied to non-invasive theranostic of desmoplastic tumors. According to the information presented by vivo imaging, such as the biodistribution and accumulation of therapeutics-loaded exosomes and real-time tumor delineation, physicians are able to easily diagnose and monitor the treatment response of desmoplastic tumors. Fluorescent dyes, fluorescent proteins, metal nanoparticles and radioactive isotopes are common imaging agents carried by exosomes.

PKH26, DiR and cyanine are the most commonly used fluorescent dyes for labeling engineered exosomes [22, 101, 121]. Jing et al. reported that cancer-derived exosomes labeled with cyanine 7 can be used as near-infrared fuorescence imaging of colon cancer to provide a real-time tumor delineation [122]. Genetically encoded reporters of fluorescent proteins (e.g., red/green fluorescent protein and antares2) are commonly attached to transmembrane proteins (e.g., CD63, CD9 and CD81) of engineered exosomes [22, 121, 123]. For instance, prostate cancer-derived exosomes loaded with antares2-CD63 demonstrated their homing property for targeting desired organs and tissues [123]. Additionally, metal nanoparticles (such as gold-iron oxide nanoparticles, superparamagnetic iron oxide nanoparticles and gadopentetate dimeglumine) as contrast agents also have been used as magnetic resonance imaging in vivo [121, 124, 131]. Bose et al. developed exosomes that carry both therapeutic agent (anti-miR-21) and imaging agent (gold-iron oxide nanoparticles) for better breast cancer theranostics [124]. Among them, anti-miR-21 exert anti-tumor effect by reducing doxorubicin resistance, gold-iron oxide nanoparticles exhibit excellent magnetic resonance imaging that achieves the precisely track the biodistribution of anti-miR-21-loaded exosomes and tumor-specific accumulation. In addition to imaging, gold-iron oxide nanoparticles can also selectively damage tumor cells via thermal ablation [125]. The study exhibits that gold-iron oxide nanoparticles have an efficient photothermal effect in 4T1 cells. Furthermore, vivo imaging of exosomes also can be achieved by radioactive isotopes, such as ^99m^Tc, ^131^I and ^111^In-oxine [101, 121]. Generally, radiolabeling exosomes are more sensitive and give more precise information than the mentioned above imaging methods. Jing et al. showed that cancer-derived exosomes labeled with ^99m^Tc can be used as nanoprobes in single-photon emission computed tomography of colon cancer to help physicians precisely obtain the biodistribution of exosomes [122].

## Conclusions and perspectives

CAFs, as the most abundant of all stromal cells merged in tumor tissues, play critical role in mediating desmoplastic tumors progression, which can not only remodel ECM to establish pathological barriers that hinder drug delivery and immune cells infiltration, but also secret abundant cytokines and ECM proteins to promote immunosuppression and CSCs-mediated drugs resistance. In this review, aiming at the significance of CAFs in tumors desmoplastic microenvironments and the complexity of microenvironment, we elaborate that engineered exosomes as promising delivery platform or directly applied for theranostic purpose can target CAFs mediated microenvironments and overcome pathological barrier to exert precise and efficient therapy of desmoplastic tumors.

Although exosomes have been applied in clinical trials, there are still some challenges need to be overcome. The first challenge for clinical use is the isolation of large amount of high purity exosomes. Traditional ultracentrifugation is unmatched with massive production of exosomes due to the low purity and time consuming [15, 22]. Some isolation methods for massive production, such as size-exclusion chromatography and polymer precipitation have been developed, but they have some disadvantages, such as the expensive equipment and low purity, respectively [132, 133]. Thus, the selection of the most suitable isolation methods will be crucial for cancer treatment. The second challenge is the high loading efficiency of exosomes. Theranostic agents could be loaded into exosomes either before or after exosome isolation. Cell transfection of peptides, proteins and RNA, is most commonly used method for the pre-isolation cargo loading of exosomes, but has the disadvantage of cytotoxicity and difficult purification [15, 16, 90]. Post-isolation loading methods of exosomes mainly contain co-incubation, electroporation and extrusion. Among them, electroporation and extrusion have a high loading efficiency, but they have the disadvantages of cargo aggregation and the probable damage of exosomal membrane, respectively. Thus, more suitable approach should be charily selected based on physicochemical properties of cargo to achieve maximum theranostic efficiency in the future.

Finally, it cannot be ignored that except for targeted delivery vehicles for desmoplastic tumors treatment, exosomes contents have been considered as potential biomarkers for early cancer theranostic prediction, for that they can inherent various information from donor cells and be non-invasively obtained from biological fluids, including blood, urine, saliva and tears [15, 21, 91, 134]. Therefore, a combination of in vitro and vivo applications of exosomes may be likely to burst out power potential in clinical theranostic, which not only can be applied for early diagnosis of cancers, but also can target CAFs mediated desmoplastic microenvironments to treat cancers and real-time monitor the treatment response, providing the next generation personalized nano-drugs for desmoplastic tumors therapy.

## Data Availability

Not applicable.

## Abbreviations

CAFs: Cancer-associated fibroblasts
ECM: Extracellular matrix
CSCs: Cancer stem cells
CSF1: Colony-stimulating factor 1
FGF5: Fibroblast growth factor 5
EMT: Epithelial mesenchymal transition
TGFβ: Transforming growth factor-β
IL-6: Interleukin-6
PDGFR: Platelet-derived growth factor receptor
SDF1: Stromal derived factor 1
HGF: Hepatocyte growth factor
FAP: Fibroblast activation protein
PDGFRs: Platelet-derived growth factor receptors
α-SMA: Alpha smooth muscle actin
CXCL: C-X-C chemokine ligand
myCAFs: Myofbroblastic CAFs
iCAFs: Infammatory CAFs
Lox: Lysyl oxidase
PDPN: Podoplanin
TPM: Tropomyosins 1
POSTN: Periostin
MMP: Matrix metallopeptidase
HAS: Hyaluronan synthases
MHC II: Major histocompatibility complex class II
TAM: Tumor associated macrophage
MDSCs: Myeloid-derived suppressor cells
CCL2: C–C chemokine ligand 2
CTLs: Cytotoxic T lymphocytes
IGF-II: Insulin-like growth factor-II
ESCRT: Endosomal sorting complex required for transport
siRNA: Small interfering RNA
miRNA: MicroRNA
DSPE-PEG: Distearoyl phosphoethanolamine-polyethylene glycol
